# Map-A-Mole: Greenspace Area Influences the Presence and Abundance of the European Mole *Talpa europaea* in Urban Habitats

**DOI:** 10.3390/ani10061097

**Published:** 2020-06-25

**Authors:** Mark D.E. Fellowes, Kojo Acquaah-Harrison, Fabio Angeoletto, Jeater W.M.C. Santos, Deleon da Silva Leandro, Elise A. Rocha, Tara J. Pirie, Rebecca L. Thomas

**Affiliations:** 1 People and Wildlife Research Group, School of Biological Sciences, Harborne Building, University of Reading, Whiteknights, Reading, Berkshire RG6 6AS, UK; kojoeah@live.co.uk (K.A.-H.); eliserocha1@gmail.com (E.A.R.); t.pirie@reading.ac.uk (T.J.P.); Rebecca.Thomas@rhul.ac.uk (R.L.T.); 2 Universidade Federal de Mato Grosso, Campus Universitário de Rondonópolis–UFMT, Rodovia Rondonópolis/Guiratinga, Rondonópolis 78736-900, MT, Brazil; fabio_angeoletto@yahoo.es (F.A.); jeatermaciel@gmail.com (J.W.M.C.S.); deleon_roo@hotmail.com (D.d.S.L.); 3 School of Biological Sciences, Royal Holloway University of London, Egham, Surrey TW20 0EX, UK

**Keywords:** urban ecology, urban planning, urban greening, urban biodiversity, mammal, habitat management, species area relationships

## Abstract

**Simple Summary:**

The European mole is a burrowing mammal which is widely distributed across Britain and much of continental Europe. Its presence is readily confirmed by the presence of molehills, which contain the spoil heaps left behind as the mole digs its underground tunnels. Despite being easy to record, there are very few studies of moles in an urban environment. We asked how area of greenspace (largely parks, recreation areas, nature reserves and playing fields), distance to the nearest patch, human disturbance, how long the green patch had been isolated for, and degree of urban construction around the patch affected mole presence and abundance. We found that patch size affected mole presence, with a minimum greenspace of approximately 10 ha required. Where moles were found, larger patches had more signs of moles and surprisingly, mole abundance was also associated with the degree of urban construction around the greenspace. This result shows how urban planning can affect the presence of unusual species, such as the European mole.

**Abstract:**

The European mole *Talpa europaea* is common across much of Britain. It has a unique fossorial lifestyle, and evidence of its presence is readily identified through the presence of characteristic molehills. Although molehills are often a common sight in urban greenspaces, moles are remarkably understudied, with very few studies to date exploring the urban ecology of moles. Here, we investigate if factors such as greenspace (largely urban parks and playing fields) area, intensity of management, distance to nearest patch, amount of time the patch had been isolated from other green patches, and the amount of urbanization (constructed surfaces) surrounding the patch, influence the distribution and abundance of urban moles. Mole signs (hills and surface runs) were counted in all discrete urban greenspaces (excluding domestic gardens and one private golf course) within an 89.5 km^2^ area in the UK town of Reading. We found that 17 out of 59 surveyed sites contained moles, with their presence being recorded in greenspaces with a minimum patch area of approximately 0.1 km^2^ (10 ha). Where present, the abundance of mole territories in the greenspaces was associated with both the area of greenspace and degree of urbanization within 150 m of the patch boundary. While the former was not surprising, the latter outcome may be a consequence of sites with an increased risk of flooding being home to fewer moles, and the surrounding area is also less likely to be built upon. This case study highlights how choices made in designing urban green infrastructure will determine which species survive in urban areas long into the future.

## 1. Introduction

Increased urbanization is associated with the destruction of natural habitats [1,2,3]. Urbanization transforms environments, most notably through the use of impervious surfaces associated with roads and building, and its effects are far-reaching for natural ecological systems [4]. Indeed, in some countries, urbanization has been found to threaten more species than any other human activity [5]. Urbanization reduces and alters environments, resulting in habitat fragmentation and isolation, with these effects reducing the abundance and diversity of local species [6,7,8] and changing patterns of ecological interactions [9,10]. In particular, transport infrastructure for roads and railways can cut through habitats [11,12,13], leading to the separation and isolation of animal populations [14]. The presence of transport networks causes a barrier effect, as many animal species avoid such structures due to disturbance from vehicles, the road surface itself and transport emissions [14,15]. This fragmentation eventually leads to eventual local extinction [14].

Some species are able to persist and even thrive in these altered habitats [16], but for most, urban areas are challenging and ecological interactions change. Urbanization in general decreases species richness for the majority of biotic communities. Nevertheless, this statement needs to be nuanced. A notable exception to this pattern is the richness of plant species in urban ecosystems, which is usually high [17]. Likewise, the biomass of birds and arthropods is sometimes greater in cities than in their rural surroundings [8]. In some cities, bird species richness can be high, and this includes endangered species [18,19]. Therefore, the different rates do not respond in the same way to urbanization and human influence on urban ecosystems. Cultural factors such as the habit of keeping birdseed dispensers for birds in backyards [20,21], or the maintenance of green areas for the practice of *candomblé*, an Afro-Brazilian religion, increase support for urban biodiversity [22]. On the other hand, relatively few species of mammals have successfully established themselves in urban ecosystems [23].

Surprisingly, there is one relatively common mammal species found in some urban locations, one whose presence is readily assessed, but which has to our knowledge received little attention from urban ecologists—the European mole (*Talpa europaea*) [24]. This species is the only fossorial mammal in Britain and holds a unique niche within its environment [24,25]. The European mole is widespread in Britain, with an estimated population of 41 M [26], but is absent from the rest of the British and Irish Isles [27]. British European moles are of the nominate subspecies (*T. europaea europaea* Linnaeus, 1758) and are most closely related to populations from northern France and Germany [28]. They inhabit a wide range of habitats and are more common in lowland grasslands, deciduous woodland and arable farmland [29,30]. They are restricted by highly acidic soils which have low densities of their most important prey species, earthworms [31,32,33,34], and they also avoid waterlogged, stony and sandy soils [31,32,33,34]. Moles are present in urban areas, as evidenced by field signs and their presence in the diet of urban tawny owls [35].

Although moles are rarely seen, mole presence is evident through their visible molehills [29,30]. European moles are territorial, and this allows estimates of territory number to be calculated, although the number of individual moles in each territory may vary [30,31]. European moles generally burrow less than a metre below the ground, but this tunnelling behaviour brings them into conflict with people, and moles are seen by some as agricultural and horticultural pests [26,34]; as a result, capturing and killing moles is legally permissible in Britain [25,36,37].

In this study we asked what factors influenced the presence of urban moles. By recording and aging mole signs, we investigated if greenspace area, distance to nearest suitable habitat, length of isolation (years that the area had been enclosed by urban development), or degree of urbanization affected the distribution and abundance (number of territories) of moles in a large urban area.

## 2. Materials and Methods

### 2.1. Study Sites

Reading is a large urban area in the southeast of England, approximately 30 km west of Greater London. The enclosed area surveyed was bounded to the north by the River Thames, to the south by the M4 motorway, and to the east and west by major roads, providing an area of c. 89.5 km^2^. The survey area is located within the British National Grid reference squares: SU 66, SU 67, SU 76 and SU 77.

Preliminary field work and discussions with residents revealed no evidence of the presence of moles in domestic gardens, but other studies have reported the presence of moles in domestic gardens in national surveys [36]. While this possibility remains (and gardens may provide links between patches as juvenile moles disperse), urban garden habitat is not considered further here. Areas of greenspace (defined by contiguous area) such as parks, playing fields, recreation grounds, woodland, and nature reserves in the area were identified using QGIS software [38] and area of each site calculated. These sites are largely natural and not the result of conversion of brownfield areas into parkland, so levels of anthropogenic materials such as rubble in the soils will generally be limited.

**Figure 1:**
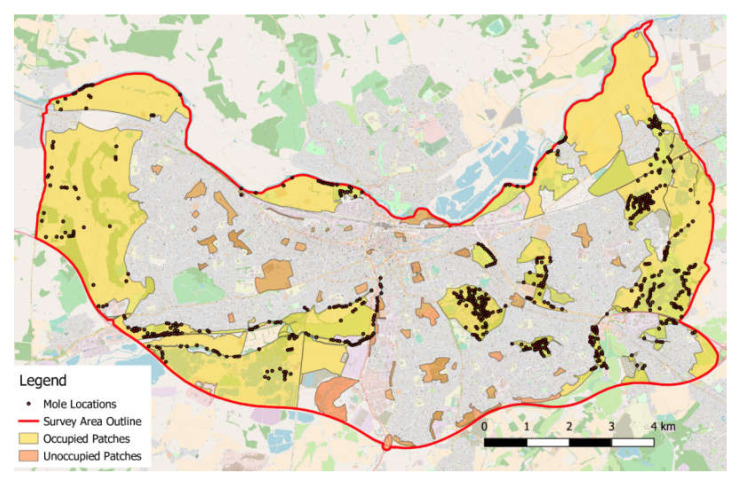
OS OpenStreetMap showing the survey area (solid boundary line) with the mole locations (filled circles) and individual occupied and unoccupied areas of open land (filled yellow and brown respectively).

Each site was visited and boundaries walked to ensure that patches were not connected to other green areas. All significant greenspaces (i.e., larger than 0.45 ha) within the study area (Greater Reading south of the River Thames, and bounded to the east, south and west by primary roads (see Figure 1 for details) were surveyed with the exception of one private golf course, which did not give permission for access.

### 2.2. Mole Surveying

Mole surveying was carried out on 74 days between 18 April and 1 July 2018. Physical signs of mole activity were searched for and location recorded using a GPS device (Garmin GPS MAP 64S). The signs used to identify their presence were the mole hills and surface runs (following 25). Recordings were made at intervals of at least 50 m and individual signs of activity were assigned to a point if they were within 25 m of the recording point. As moles are territorial, each cluster was considered to be independent [30]. Where possible, recordings were made at the most prominent, centralised signs in a cluster of molehills. However, to ensure that signs were not unnecessarily missed, less prominent signs were also used as the record point as long as they were the correct distance from the other recording points.

In the smaller patches within the town’s limits, the entire area was surveyed so long as public access allowed it, whereas in larger patches, transects were surveyed across them that were laid alongside public footpaths, rights of way, roads and rivers, covering as much of the accessible space as possible. It is therefore possible that moles were under-recorded in larger areas of greenspace where access was not possible. For presence/absence data, our chances of a false negative (recording moles as being absent from a site, when they were present) or a false positive (recording moles as present, when actually absent) errors is low, as all accessible suitable habitat in sites was surveyed, and mole signs are highly distinctive.

Fifty metres was used as the minimum recording interval distance between mole hills based on the habitat types of the areas being surveyed. A large amount of the area covered in the patches surveyed was made up of grassland, and previous studies have indicated that mole territories, while varying in size, are often smaller in habitats like grassland due to the higher prey densities [30]. To develop the survey technique, preliminary work was trialled on the University of Reading Whiteknights campus by recording every mole sign that was discovered between 18 April 2018 and 5 May 2018. It was found that although some signs would have to inevitably be unrecorded due to their distance from the main recording points, a 50 m interval allowed for the great majority to be recorded.

### 2.3. Other Variables

Besides mole presence/absence and the number of mole recordings, the other variables examined included the area of the patches in kilometres squared, how many years a patch had been isolated, and the distance to the nearest suitable patch in metres.

The study also looked at the intensity of human management within a patch using a grading scale one to three; from heavily managed to little or no management. Evidence of human management and land use activities such as scrub clearance, ploughing, planting and mowing were used to determine the level of management intensity across a patch. As an added measure, requests for information were emailed to West Berkshire council, Reading Borough Council and Wokingham Borough Council. They were asked if they had any information regarding control measures used to remove moles within the parts of the survey area that they look after.

Finally, the proportion of constructed surface within three buffer zones set at 50, 100 and 150 m from the patch itself was used to determine the degree of urbanization around the site, with more urbanized sites being surrounded by proportionally more constructed surfaces. Estimates of the number of years isolated were determined by analysing old OS Survey maps with known production dates and identifying the first map where a green patch could be identified as being completely isolated from all other patches.

### 2.4. GIS Data Analysis

QGIS software [38] was used to carry out all GIS analysis which began by converting all the mole sign grid references recorded into their corresponding X and Y co-ordinates. The coordinates were then imported into QGIS where they could be converted into points in a shapefile layer. The size of each mole patch was determined and measured by creating a shapefile layer of polygons and placing them over the top of an OS Base map layer. The patches themselves were defined based on connectivity; for example a flattened bridge or underpass that a mole could potentially cross would connect two areas that are divided by a river and thus those areas would be considered to be part of one patch. Similarly, areas that were surrounded by sizable enough barriers, such as a railway line or clusters of buildings, would be counted as separate from the areas just beyond the aforementioned barriers. The measurement tool and identify tool were used to calculate the distances between the nearest patches and the area of the individual patches, respectively. To work out the proportion of constructed surfaces surrounding the patches, the multi-ring buffer tool was used to create three separate buffer zones around each polygon; the measurement tool could then be used to work out the constructed surfaces area by drawing polygons over these areas in a Google Satellite map layer allowing the proportion of constructed surface surrounding the patch to be calculated.

### 2.5. Statistical Analysis

All analyses were performed using the R3.1.2 [39]. Analysis with different buffer sizes of constructed surfaces were done separately due to the high correlation between these variables. The group of explanatory factors predicting the response variables was tested through variance inflation factors (VIF values) [40], and all the range of VIF values were below 3. Second order interactions between the explanatory variables distance to the nearest patch, proportion of constructed surface, years isolated and area were also explored for any significant interaction term.

The response variable presence (or absence) of moles was analysed through a logistic generalised linear model with logit link (package stats [39]). Data exploration for outliers in the explanatory variables revealed the need for the transformation of patch/area, which was log transformed.

Mole counts were analysed through a generalised negative binomial model with log link (package MASS [41]). Data exploration for outliers in the explanatory variables revealed the need for transformation of Patch/Area (log10 transformed), years isolated (square root transformed), and distance to the nearest patch (log10 transformed).

Model simplification was done by applying the automatic stepwise model procedure using Akaike Information Criterion (AIC) comparisons between nested models using the function stepAIC (package MASS [41]). Models were investigated for potential temporal and spatial serial autocorrelation by the Durbin–Watson test (package car [42]). The validity of all models was investigated by evaluating the normality, homogeneity and independence of model residuals.

## 3. Results

### 3.1. Surveying Results

The surveys found 650 mole records (territories) throughout the study area. Fifty-nine separate patches were defined based on the areas visited and 17 of these patches were found to be occupied by moles.

### 3.2. Statistical Analysis

**Figure 2:**
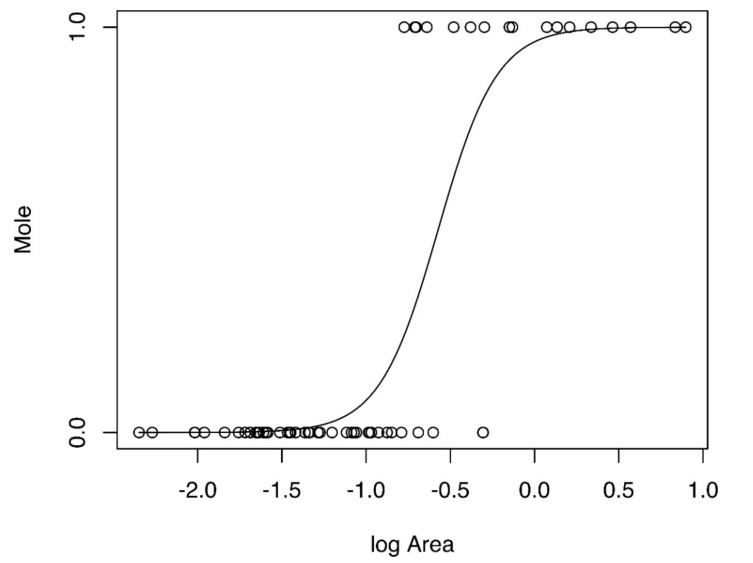
Representation of logistic regression model of patch/log area (km^2^) predicting the presence (1) or absence (0) of moles. Note that multivariate logistic regression models were performed; however, just one significant explanatory variable is present here to illustrate the direction of relationship between variables.

**Figure 3:**
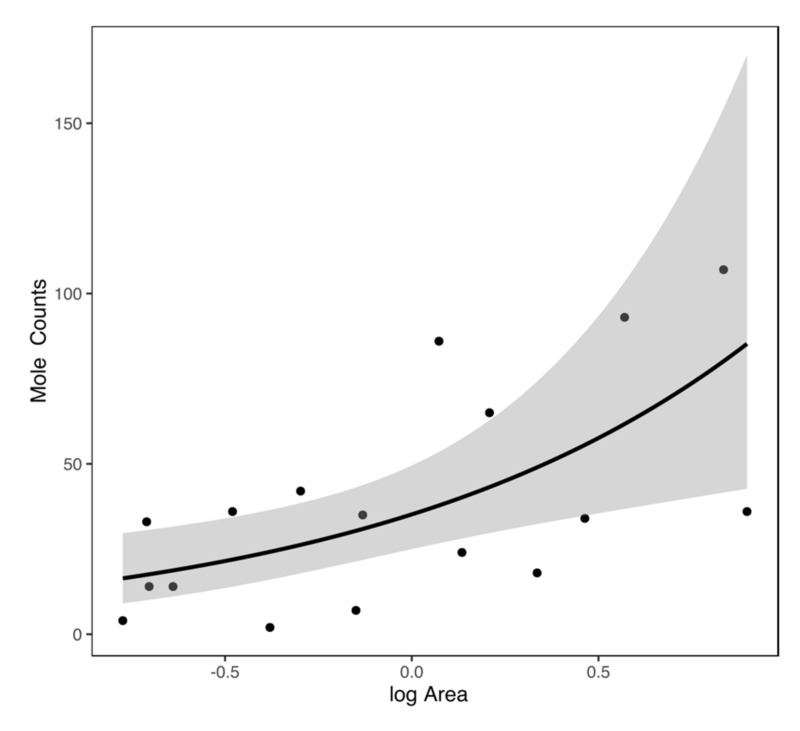
Representation of the generalised negative binomial model of patch/log area (km^2^) predicting mole counts. Note that multivariate negative binomial models were performed; however, the trend line of just one significant explanatory variable is presented here to illustrate the direction of the relationship between variables. Mole counts are the number of separate mole patches (territories) recorded in each location.

**Figure 4:**
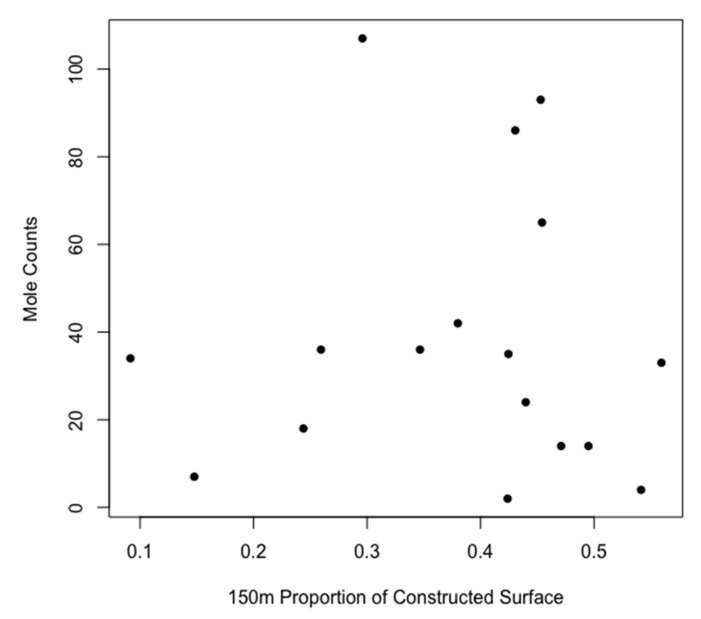
Numbers of mole territories related to the proportion of constructed surface in a 150 m buffer zone around urban greenspaces occupied by moles.

For all three buffer zones, mole presence was significantly and positively determined by the area of each patch (Table 1, Figure 2), and examination suggests that a minimum patch area of greater than ~10 ha is required for mole presence. For mole abundance in buffers of 50 and 100 m, only the area of the patch predicted mole counts significantly. For a buffer zone of 150 m, both the patch area and the proportion of constructed surface predicted mole abundance positively and significantly in occupied patches (Table 2, Figure 3 and Figure 4). No other factors were significant.

## 4. Discussion

Moles are a surprisingly understudied species, given how common and widespread they are. To our knowledge, this is the first study of what limits the presence and abundance of the European mole in urban habitats. Our work revealed that moles were widespread in urban, suburban and periurban greenspaces in our study area, but their presence was determined by patch size, with a minimum patch size of greater than approximately 10 hectares required for the presence of moles. For occupied patches, the counts of mole signs were positively associated with patch area. We found no effect of length of isolation of green areas on mole presence (although we note that only two patches enclosed by urban development more than 56 years ago were inhabited by moles). There was no relationship between the intensity of urbanization (measured as the proportion constructed surfaces within a 150 m boundary of the greenspace) and the presence of moles. Surprisingly, in sites where moles were present, mole population size as estimated by mole territories, was higher in areas with more surrounding constructed areas.

The relationship between the presence/absence of species and area of urban greenspace is well established. Simply put, all else being equal, larger areas of suitable habitat hold more individuals and species. Our finding that moles require a greenspace area greater than ~10 hectares to persist provides a new example of how this effect may be driven. There are a number of reasons why greenspace area may determine mole presence. The most parsimonious is that areas smaller than this may not be able to maintain a self-sustaining population size for this territorial species. However, it is also likely that area of greenspace is not independent of patterns of use, management and habitat diversity.

The visual method of surveying for mole signs was successful, allowing us to readily assess the presence or absence of moles using simple census techniques [43,44,45]. However, we note that there are challenges in using this approach, as molehills may not always be visible (particularly in less accessible areas) where leaf litter, thick undergrowth, and tall grasses could completely hide mole signs. Furthermore, moles are less likely to excavate new hills in certain conditions, such as long dry spells [29,30,31], although we chose to record before mid-summer to minimise this.

One further factor which may affect the distribution of moles is that of management, as moles can be considered a pest species for damage done to the surfaces of playing fields and recreation areas. Management in the form of constructed barricades (e.g., fences or netting driven deep into the ground) is not known in the area, and we used Freedom of Information Act requests to ask the two relevant local councils if they used any methods to control moles. Only one council reported using mole control methods in the past 18 years, and this was in a small area of one greenspace. We cannot of course reject the hypothesis that mole control has been used in the past, or by other actors, but we have no evidence that control measures are likely to have affected our results, and indeed the widespread distribution of moles in our study region suggests that any control measures will have been minimal, notwithstanding their pest status [36].

Where moles were present, mole abundance (indirectly measured as the number of molehill clusters) was positively correlated with greenspace area and the proportion of constructed surfaces within 150 m of the greenspace. The former is unsurprising, e.g., [46], although a positive patch area/abundance relationship is not always found in mammals [47]. The finding that increased mole numbers were associated with the proportion of constructed surfaces within 150m is surprising and is worthy of further examination. Surveying the sites, we speculate that this may be an artifact of location, where sites that are more prone to flooding have a reduced density of constructed surfaces, and also fewer moles. This requires further examination.

We note that our estimates of abundance are indirect, and are perhaps best considered as a measure of the intensity of mole presence, as territories will have multiple individuals, and we may have made errors in assigning mole signs to a territory. Studies have been able determine accurate territory boundaries by live trapping moles before attaching radio transmitters or radioactive rings to the animals, allowing for tracking in real time [48,49]. While estimating population sizes (as opposed to counting estimated territory numbers) would have been of considerable interest, it necessarily would be limited to small patches, and so is not logistically feasible for studies such as ours. Nevertheless, this method does provide very useful insights into mole numbers.

The European mole is a very well-known species, playing a role in many cultures. Their presence is readily confirmed, and they are a visible part of urban ecosystems across much of Europe. Despite this, they have not been studied by urban ecologists. We suggest that this species would reward further consideration.

## 5. Conclusions

Urbanization profoundly alters landscapes, and consequently the presence and abundance of the species that are able to tolerate these highly managed and disturbed systems. The European mole is a species that is directly influenced by the management strategies adopted by people in these landscapes, and here we find that moles were positively influenced by the size of the area surveyed. For mole populations to be able to survive, greenspaces greater than approximately 10 ha are needed, and where present, population size increases with greenspace area, and unexpectedly with the degree of urbanization around the site. Being subterranean, moles are perhaps less influenced by factors such a human disturbance, but they will be sensitive to land management strategies, especially those, such as fragmentation by roads and development, that will influence the ability of juveniles to disperse. Considered in some quarters to be a pest species, moles contribute towards the health of the soil ecosystem and should be considered when formulating development plans in urban development. This study also illustrates that this species, which is widespread and well-known where present, is surprisingly poorly studied, and is worthy of further attention.

## Figures and Tables

**Table 1:** Summary of the best logistic regression models predicting the presence or absence of moles for each buffer size. AIC values for each model are given.

Mole Presence				
Buffer Size	Explanatory Variable	Coefficient Value ± SE	*p*	AIC
50 m	Intercept	3.288 ± 1.286	0.010	27.291
	logArea	5.742 ± 1.752	0.001	
100 m	Intercept	9.194 ± 4.706	0.051	26.286
	logArea	5.312 ± 1.930	0.006	
	100 m CS	−12.846 ± 8.603	0.135	
150 m	Intercept	8.041 ± 4.217	0.056	26.889
	logArea	5.235 ± 1.791	0.003	
	150 m CS	−10.443 ± 7.676	0.174	

**Table 2:** Summary of the best negative binomial models predicting the numbers of moles detected on each buffer zone. AIC values for each model are given.

Mole Counts				
Buffer Size	Explanatory Variable	Coefficient Value ± SE	*p*	AIC
50 m	Intercept	3.560 ± 0.174	0.000	156.43
	logArea	0.986 ± 0.331	0.003	
100 m	Intercept	3.560 ± 0.174	0.000	156.43
	logArea	0.986 ± 0.331	0.003	
150 m	Intercept	2.919 ± 0.558	0.000	156.39
	logArea	1.035 ± 0.343	0.002	
	logDistance NP	−0.417 ± 0.252	0.098	
	150 m CS	3.090 ± 1.483	0.038	
